# Point-of-Care/Chairside aMMP-8 Analytics of Periodontal Diseases’ Activity and Episodic Progression

**DOI:** 10.3390/diagnostics8040074

**Published:** 2018-10-22

**Authors:** Ismo T. Räisänen, Anna Maria Heikkinen, Eva Siren, Taina Tervahartiala, Dirk-Rolf Gieselmann, Gerrit-Jan van der Schoor, Peter van der Schoor, Timo Sorsa

**Affiliations:** 1 Department of Oral and Maxillofacial Diseases, Head and Neck Center, University of Helsinki and Helsinki University Hospital, PO Box 63 (Haartmaninkatu 8), 00014 Helsinki, Finland; anna.m.heikkinen@helsinki.fi (A.M.H.); eva.siren@helsinki.fi (E.S.); taina.tervahartiala@helsinki.fi (T.T.); 2 Institute for Molecular Diagnostics (IMOD), Bonner Str. 84, 42697 Solingen, Germany; gieselmann@matrix-lab.de; 3 Division of Periodontology, Department of Dental Medicine, Karolinska Institute, SE-171 77 Stockholm, Sweden; vdschoor.putten@gmail.com (G.-J.v.d.S.); peter@vanderschoor.org (P.v.d.S.); timo.sorsa@helsinki.fi (T.S.)

**Keywords:** matrix metalloproteinases, periodontal diseases, periodontitis, point-of-care testing

## Abstract

Traditional periodontal disease diagnostics are based mainly on clinical examination and radiographs. They assess only past tissue destruction and provide no information on the current disease status or its future progression. The objective is to find out if an active matrix metalloproteinase-8 (aMMP-8) point-of-care (PoC) test could provide a cost-effective way to get around this limitation. This cross-sectional study used 47 adolescents and 70 adults, who were clinically examined and their aMMP-8 PoC tested. The aMMP-8 PoC test results and patients’ treatment need, based on the community periodontal index of treatment needs (CPITN), were compared and analyzed using Fisher’s exact test. In terms of CPITN, the aMMP-8 PoC test gave no false positives for both adolescents and adults. All healthy patients got a negative test result, while a positive test result indicated periodontal treatment need correctly. Finally, there was a significant association between a patient’s aMMP-8 PoC test result and his/her treatment need (*p* = 0.001 for adolescents, *p* = 0.001 for adults). In conclusion, more accurate diagnostics of periodontal diseases’ activity and progression using an aMMP-8 PoC test may help to reduce oral health care costs by reducing patient overtreatment, improving patient outcome, and reducing the need for complex periodontal therapy.

Diagnosis of periodontal diseases is traditionally based on clinical examination and periodontal parameters that include probing depths, bleeding on probing, clinical attachment level, and radiographs (alveolar bone loss). Currently, this is considered the best available method for diagnosing and monitoring the disease’s course, treatment response, and maintenance therapy period [1]. Unfortunately, these traditional diagnostic methods offer only a retrospective evidence of the destructive disease process, as well as its current extent and severity [1]. They offer no reliable information on the periodontal diseases’ current activity and its episodic progression [1]. Thus, there is a real need to find new methods to get around this limitation. It complicates not only the prediction of the future disease progression but also the targeting and the timing of the prevention of periodontal diseases. Furthermore, it makes the monitoring of the treatment response and maintenance therapy somewhat imprecise, as the activity of disease progression measured before/after periodontal treatment (e.g., scaling and root planing) is only retrospective. Moreover, there exist some promising treatment modalities that may help to reduce the activity and episodic progression of periodontal diseases [2,3,4]. Monitoring their effects would also benefit from this kind of information.

Neutrophil collagenase, also known as matrix metalloproteinase-8 (MMP-8), has been identified as a major collagenolytic enzyme that causes active periodontal tissue destruction in periodontitis and peri-implantitis [5,6]. A key characteristic of active periodontal diseases is the pathological elevation and activation of MMP-8 to active MMP-8 (aMMP-8) in periodontal tissues, which are reflected in oral fluids (e.g., saliva, mouth rinse, gingival crevicular fluid, and peri-implant sulcular fluid) [6,7,8]. There exists a quantitative point-of-care (PoC) lateral flow collagenase-2 (aMMP-8) oral fluid test that has, repeatedly and independently, been validated among both adults and adolescents in different ethnic populations, as a reliable tool to target periodontal diseases, with a turnaround time of only 5–7 min [6]. In this study, our aim was to assess if an aMMP-8 PoC test could provide a cost-effective way to evaluate the current disease activity and the future progression of periodontal diseases.

Figure 1 demonstrates the distribution of the aMMP-8 PoC test results according to patients’ treatment need, based on the community periodontal index of treatment needs (CPITN) [9,10]. Our sample consists of 47 adolescents (aged 15–17, described by Heikkinen et al. [11]) and 70 adults (aged 27–88, collected from the database of 15,000 patients of Finnish dental health care company called Oral Hammaslääkärit). Their periodontal disease was characterized clinically and with X-ray analysis, as described earlier [7,11,12,13,14,15]. Furthermore, CPITN scores in Figure 1 include patients’ clinical periodontal characteristics (i.e., frequency of patients with and without bleeding on probing, root calculus, and ≥4 mm periodontal pockets).

In terms of CPITN, the aMMP-8 PoC test gave no false positives for both adolescents and adults. In other words, all healthy patients (CPITN = 0 to 1) were classified correctly and had a negative aMMP-8 PoC test result. A positive aMMP-8 PoC test result occurred only if patients were in need of periodontal treatment (CPITN = 3 for adolescents, CPITN = 2–4 for adults) (Figure 1). Also, there were some patients that had no active periodontal tissue destruction in progress (a negative aMMP-8 PoC test result), while they needed periodontal treatment (CPITN = 3 for adolescents, CPITN = 2–3 for adults) (Figure 1). Thus, they were not at risk for the progression of periodontal diseases at that moment. However, none of the test negative patients had any deep (≥6 mm) periodontal pockets. Furthermore, Fisher’s exact test revealed a significant association between a patients’ aMMP-8 PoC test result and his/her treatment need (*p* = 0.001 for adolescents, *p* = 0.001 for adults).

Currently, there exists a visual and a quantitative aMMP-8 PoC test that is available for the diagnostics of the active periodontal tissue destruction in periodontitis and peri-implantitis [6,16]. Previously, the visual aMMP-8 PoC test has been validated in Finland, Nigeria, Germany, Holland, Malawi, Turkey, Sweden, and the USA [11,13,15,16,17,18,19,20]. The cut-off point (20 ng/mL) of the visual aMMP-8 PoC test has been defined and validated by an independent consulting company before making this test commercially available, and it has also been validated independently and globally in Europe, Africa, and the USA [11,13,15,16,17,18,19,20]. In this study, we used the visual aMMP-8 PoC test and no mouth rinse samples were collected for further analyses. Thus, we were not able to produce quantitative results based on them. However, there has been a clear correlation reported between the visual and the quantitative aMMP-8 PoC test result with the cut-off point (20 ng/mL) when assessing the effects of periodontal treatment in periodontitis [16].

The main result of this study was that there was a statistically significant association between a patient’s aMMP-8 PoC test result and his/her periodontal treatment need determined using CPITN. The aMMP-8 PoC test classified all healthy patients correctly (no false positives), while all test positive patients were in need for periodontal treatment. Thus, the aMMP-8 PoC test didn’t cause any patient overtreatment. We suggest that the aMMP-8 PoC test may enhance periodontal risk assessment notably and, therefore, it should be incorporated into the periodontal disease diagnostics, as well as periodontal health and the screening of treatment need. Its ability to reliably identify the patients who are at higher risk for active periodontal tissue destruction and progression of periodontal diseases is crucial for oral health care professionals trying to optimize patient treatment and improve outcomes for patients. First, the aMMP-8 PoC test may improve the targeting and the timing of the prevention of periodontal diseases. As our results demonstrate, active tissue destruction may occur not only among patients that have periodontal pockets but also among patients that do not have periodontal pockets. They both are at early risk for the development of periodontal diseases. Their treatment should be planned accordingly to minimize the periodontal tissue destruction and prevent/slow down the progression of periodontal diseases. Secondly, the aMMP-8 PoC test may aid in monitoring the treatment response and maintenance therapy and make it more accurate and precise. Oral health care professionals should aim at controlling a patient’s active tissue destruction and eliminating it with the right periodontal treatment modalities. Finally, we believe that combining an aMMP-8 PoC test with the traditional periodontal diagnostics provides more comprehensive information about patients and their periodontal disease status. It may help to reduce patient overtreatment, the need for complex periodontal therapy, and improve patient outcome, resulting in reduced oral health care costs. The quantitative aMMP-8 PoC test is inexpensive, quick, easy to use, and non-invasive diagnostic tool in comparison to traditional diagnostic methods [6,16]. It is currently available for routine use by dental and medical professionals linking these disciplines [6,16].

## Figures and Tables

**Figure 1:**
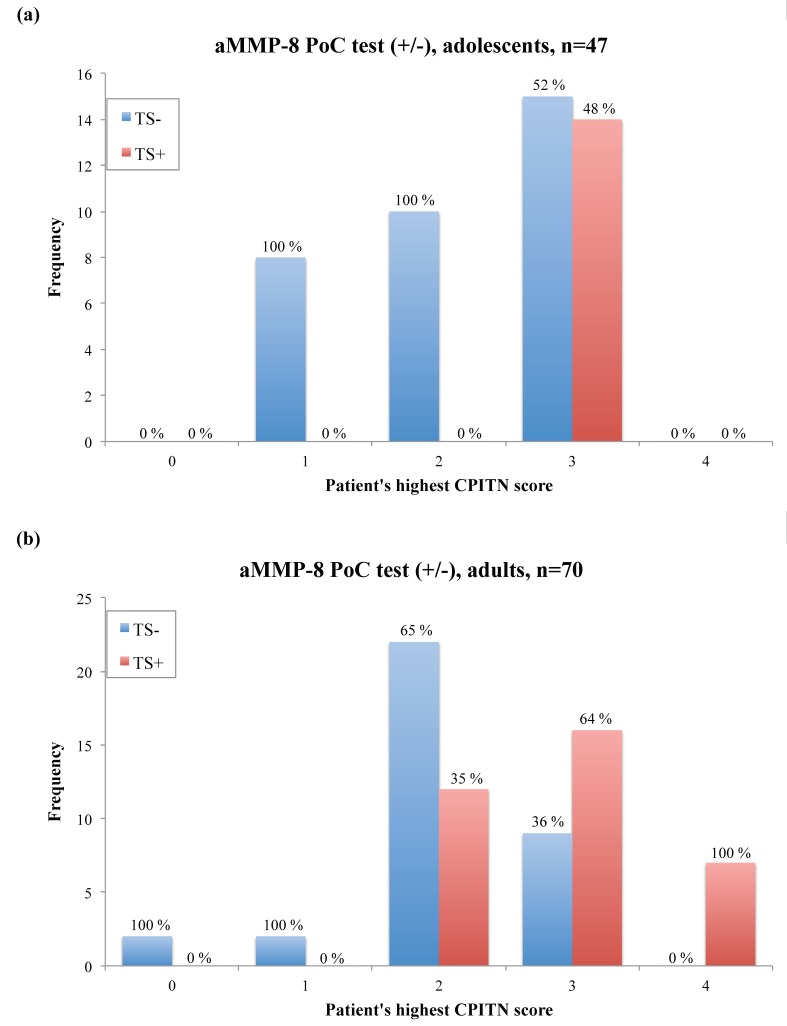
Distribution of the aMMP-8 point-of-care (PoC)/chairside test results according to patients’ treatment need, determined by a patient’s highest CPITN score found in six sextants [9,10]. Percentage of positive and negative test results per CPITN score groups is displayed over each column. (**a**) Adolescents, *n* = 47 (aged 15–17, described by Heikkinen et al. [11]). (**b**) Adults, *n* = 70 (aged 27–88, collected from the database of 15,000 patients of Finnish dental health care company called Oral Hammaslääkärit). TS− = aMMP-8 PoC test negative, TS+ = aMMP-8 PoC test positive, CPITN = community periodontal index of treatment needs.
